# Laparoscopic vs. Open Pyloromyotomy in Treatment of Infantile Hypertrophic Pyloric Stenosis

**DOI:** 10.3389/fped.2020.00426

**Published:** 2020-08-21

**Authors:** Ibrahim Ismail, Radi Elsherbini, Adham Elsaied, Kamal Aly, Hesham Sheir

**Affiliations:** Pediatric Surgery Department, Mansoura University Chlidren Hospital, Mansoura University, Mansoura, Egypt

**Keywords:** pyloric stenosis, laparoscopic, Ramstedt's, pyloromyotomy, mucosal perforation

## Abstract

**Background/Purpose:** Laparoscopic pyloromyotomy gained wide popularity in management of pyloric stenosis with contradictory results regarding its benefits over classic open approach. This study aimed at comparing both regarding their safety, efficiency, and outcome.

**Methods:** This is a prospective randomized controlled study performed from April 2017 to April 2019. It included 80 patients, divided randomly into two groups, where laparoscopic pyloromyotomy was performed in group A and open pyloromyotomy in group B. Both groups were compared regarding operative time, post-operative pain score, time required to reach full feeding, hospital stay, complications, and parents' satisfaction.

**Results:** Median operative time was 21 min in group A vs. 30 min in group B (*P* = 0). Pain Assessment in Neonates scores were generally higher in group B with more doses of analgesics required (*P* = 0). Mean time needed to reach full feeding was 15.2 and 18.8 h in groups A and B, respectively (*P* = 0). Median hospital stay was 19 h in group A and 22 h in group B (*P* = 0.004). Parents' satisfaction also was in favor of group A (*P* = 0.045). Although no significant difference was reported between both groups regarding early and late complications, some complications such as mucosal perforation and incomplete pyloromyotomy occurred in the laparoscopic group only.

**Conclusion:** Laparoscopic pyloromyotomy was found superior to open approach regarding faster operative time, less need of analgesics, easier development of oral feeding, shorter hospital stay, and better parents' satisfaction. Yet, there are still some concerns about the safety and efficiency of this procedure over open technique.

## Introduction

Infantile hypertrophic pyloric stenosis (IHPS) is a common disease in infants and occurs in about 2–4 per 1,000 live births (1). Surgical pyloromyotomy is the standard treatment for IHPS and was classically approached via an open upper quadrant or supraumbilical incision (2). Laparoscopic pyloromyotomy (LP) is gaining popularity since its introduction by Alain et al. (3) in 1991.

Laparoscopic pyloromyotomy was expected to add the advantages of the minimal invasive surgery resulting in faster recovery, shorter hospital stay, and better cosmetic outcome when compared with open pyloromyotomy (OP) (4, 5). Conversely, it was unclear whether the use of laparoscopy may lead to a higher complications rate and exposure to possible side effects of carbon dioxide insufflation in infants (6, 7). Owing to the conflicting results from randomized controlled trials, there is no clear consensus among authors and pediatric surgeons on which approach carries better results and lower incidence of complications over the other (8–10).

## Aim of the Study

The aim of this study is to compare both laparoscopic and OP techniques as regard the outcome; including operative time, time to reach full feeding, post-operative pain, and hospital stay, in addition to the rate of complications and the cosmetic outcome.

## Patients and Methods

This is a prospective randomized comparative study including 80 cases suffering from IHPS that were admitted in the pediatric surgery department at Mansoura University Children Hospital, during the period from April 2017 to April 2019. These cases were allocated randomly, by using the blocking method, into two groups: group (A): LP group, and group (B): OP group. *A priori* analysis using G^*^Power was done to estimate the study sample size. A power of 84%, type I error of 0.05 and effect size of 60% yielded a sample size of 80 cases (40 cases per group) (11).

All cases diagnosed as IHPS were eligible to be included in the study after exclusion of cases with associated anomalies such as complex congenital heart disease, which may affect the outcome, and add risks to the laparoscopic surgery.

All cases were diagnosed by clinical evaluation and abdominal ultrasonography (US) with reporting of the age at onset, age at time of operation, sex, body weight, sonographic measures of the pyloric canal and muscle, and the time needed to correct dehydration.

A signed informed consent was obtained after full counseling with the patients' parents. Group A cases underwent LP with the baby in supine position with feet at the edge of the table, and the screen is facing the surgeon at the baby's head. A 5-mm trocar was inserted through an umbilical incision for the camera port. After establishing pneumoperitoneum, the first stab incision is done on the right side below the edge of the liver just outside the midclavicular line, and a grasper is introduced to grasp the duodenum. Another left stab incision is done: either in a similar position but slightly higher when using the diathermy for pyloromyotomy or just to the left of the midline between the umbilicus and xiphisternum when using the knife, then pyloromyotomy is initiated. It is done in the avascular plane using a no. 69 blade on a small round scalpel handle or diathermy hook. The cut extends from the anterior of the stomach antrum proximally to the vein of Mayo distally. The pyloric incision is deepened, and muscle edges are spread to expose the mucosa using pyloric spreader. After completion of the procedure, hemostasis is secured; removal of ports and closure of stab incisions are performed.

On the other hand, group B cases underwent OP through an upper right transverse incision (3–4 cm). The stomach is visualized, and the pyloric tumor is identified. Without delivering the stomach, the seromuscular incision is done using a no. 15 blade knife in the avascular plane from the vein of Mayo distally to the stomach antrum proximally. A blunt instrument (mosquito forceps) is used cautiously to split the muscle edges. Perforation test was done by injection of 30 mL air via the Ryle tube and pass it gently through the pyloric canal. Hemostasis is secured, and the wound is closed in layers.

In both groups, operative time was calculated from incision to dressing, and intraoperative complications were reported. Moreover, the incidence of conversion to open approach and the causes of conversion were documented in group A.

Post-operative pain assessment started 1 h post-operatively and was guided by Pain Assessment In Neonates (PAIN) scale (12), where an analgesic dose of acetaminophen (7.5 mg/kg) is administered when the score exceeds 4. If there was no complications that require delay of feeding, feeding was started 4–6 h post-operatively according to the feeding protocol recommended by Schwartz (13) (Table 1). Every time the baby rejected his feed by vomiting was counted, and with each vomit, a period of 3-h rest was allowed, before resuming the feeding schedule from the last feed the baby should have received. Accordingly the time to reach full oral feeding was calculated and compared in both groups. It was defined as the time needed for the baby to completely tolerate his oral feeding to the full as desired with no vomiting or obstacles of any kind.

**Table 1:** Post-pyloromyotomy feeding schedule (13).

**Post-pyloromyotomy feeding schedule:**
Glucose 5% solution, 30 mL orally every 3 h ^*^1. Full-strength formula, 30 mL orally every 3 h ^*^1. Full-strength formula, 45 mL orally every 3 h ^*^2. Full-strength formula, 60 mL orally every 3 h ^*^1. Full-strength formula, 75 mL orally every 3 h ^*^1. Full-strength formula as desired. For preterm and low-body-weight infants, starting volume can be reduced to 15 mL and schedule is stopped at 60 mL.

Furthermore, both groups were compared for the time from operation to discharge. All cases were followed up for 6 months for post-operative complications. Parents' satisfaction with the cosmetic outcome was assessed 3 months post-operatively by asking parents to report their impression (very good, good, or poor).

IBM SPSS statistics (V. 25.0, IBM Corp., USA, 2017–2018) was used for data analysis. Date were expressed as mean ± SD for quantitative parametric measures in addition to median and percentiles for quantitative non-parametric measures and both number and percentage for categorized data. Student *t*-test was used for comparison between two independent mean groups for parametric data, whereas Wilcoxon rank sum test was used for non-parametric data. A χ^2^ test was used to study the association between each two variables or comparison between two independent groups as regards the categorized data. The probability of error at 0.05 was considered significant.

## Results

Both groups of laparoscopic and OP were matched regarding the age at onset, age at time of operation, sex, body weight, sonographic dimensions of the pyloric muscle, and the time needed to correct dehydration, with no statistically significant difference that may affect the comparative study.

**Figure 1:**
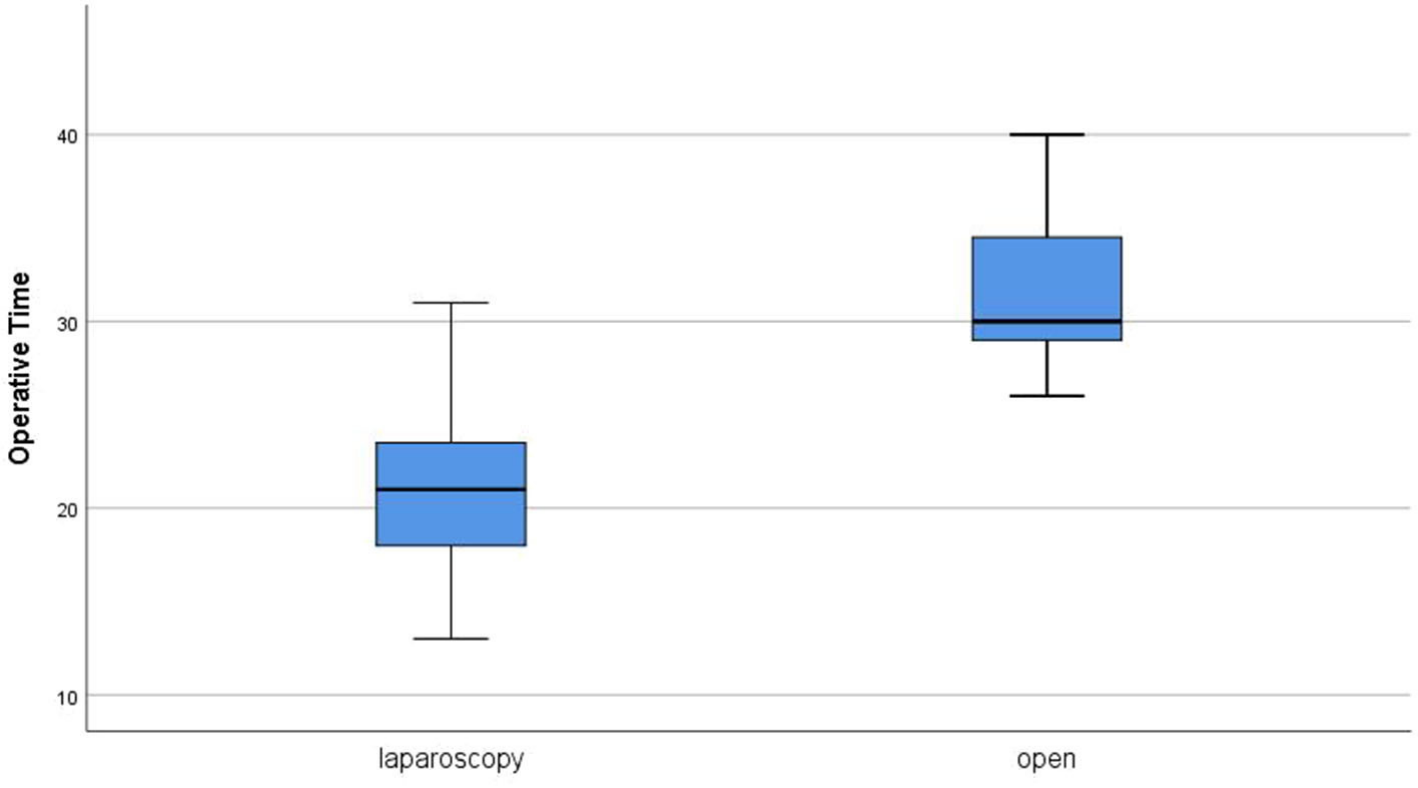
Operative time in both groups.

Operative time ranged from 13 to 75 min with a median of 21 min in group A. In group B, the median operative time was 30 min (range, 26–55 min). The statistical difference between the two groups was highly significant (*P* = 0) (Figure 1). Two cases in group A were converted to open approach (conversion rate 5%) because of mucosal perforation in one case and false suspicion of gastric injury in the other. Mucosal perforation occurred in two cases in group A (5%). It was detected during the operation in one case and repaired after conversion to open approach with omental patch and redo pyloromyotomy in another site. The other case was presented 24 h post-operatively in the same admission by persistent fever, abdominal pain, persistent vomiting, and free fluid in US examination. Similar management was performed after open exploration. Although no mucosal perforation was reported in group B, there was no statistically significant difference in comparison (*P* = 0.152). No other intraoperative complications were reported in both groups except bleeding in one case during OP and managed by cauterization of the bleeder.

**Figure 2:**
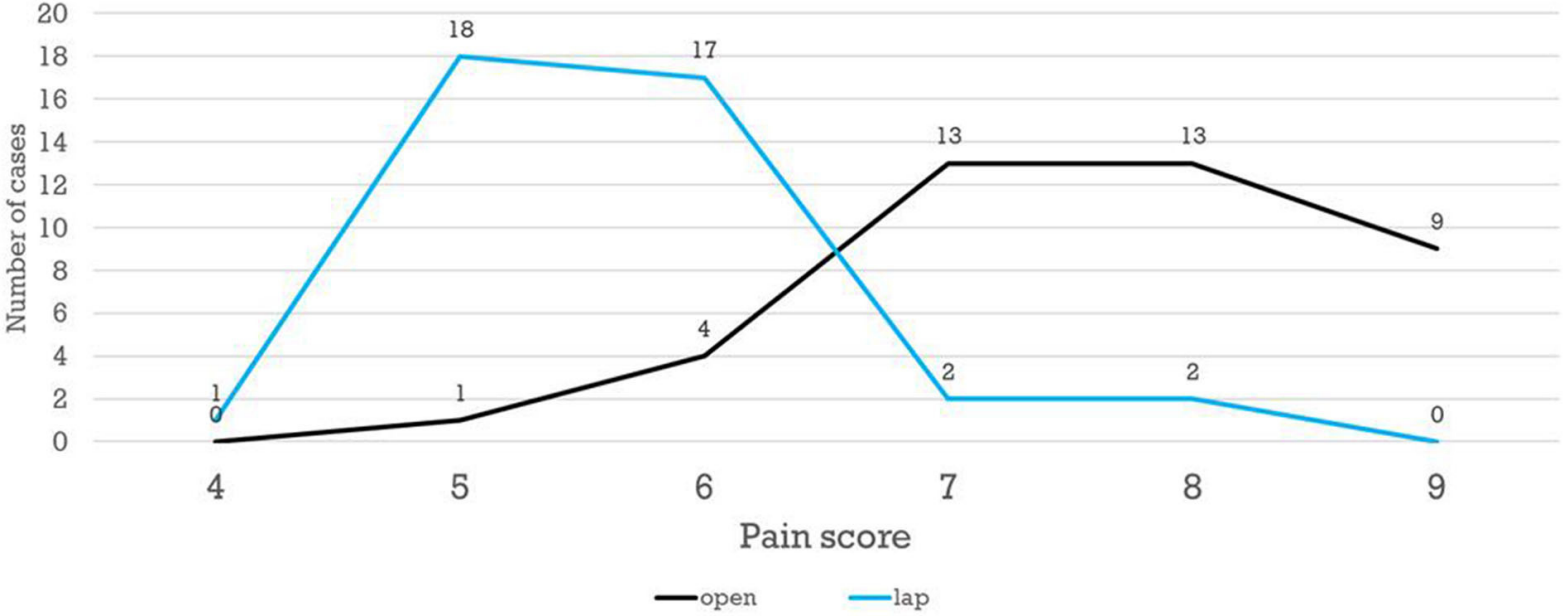
PAIN score distribution in both groups.

The patients' scores on PAIN scale ranged from 4 to 9. Patients in group B showed generally higher scores of post-operative pain after open technique; hence, more doses of analgesia were needed in comparison to group A, with a statistically significant difference reported (*P* = 0) (Figure 2).

**Figure 3:**
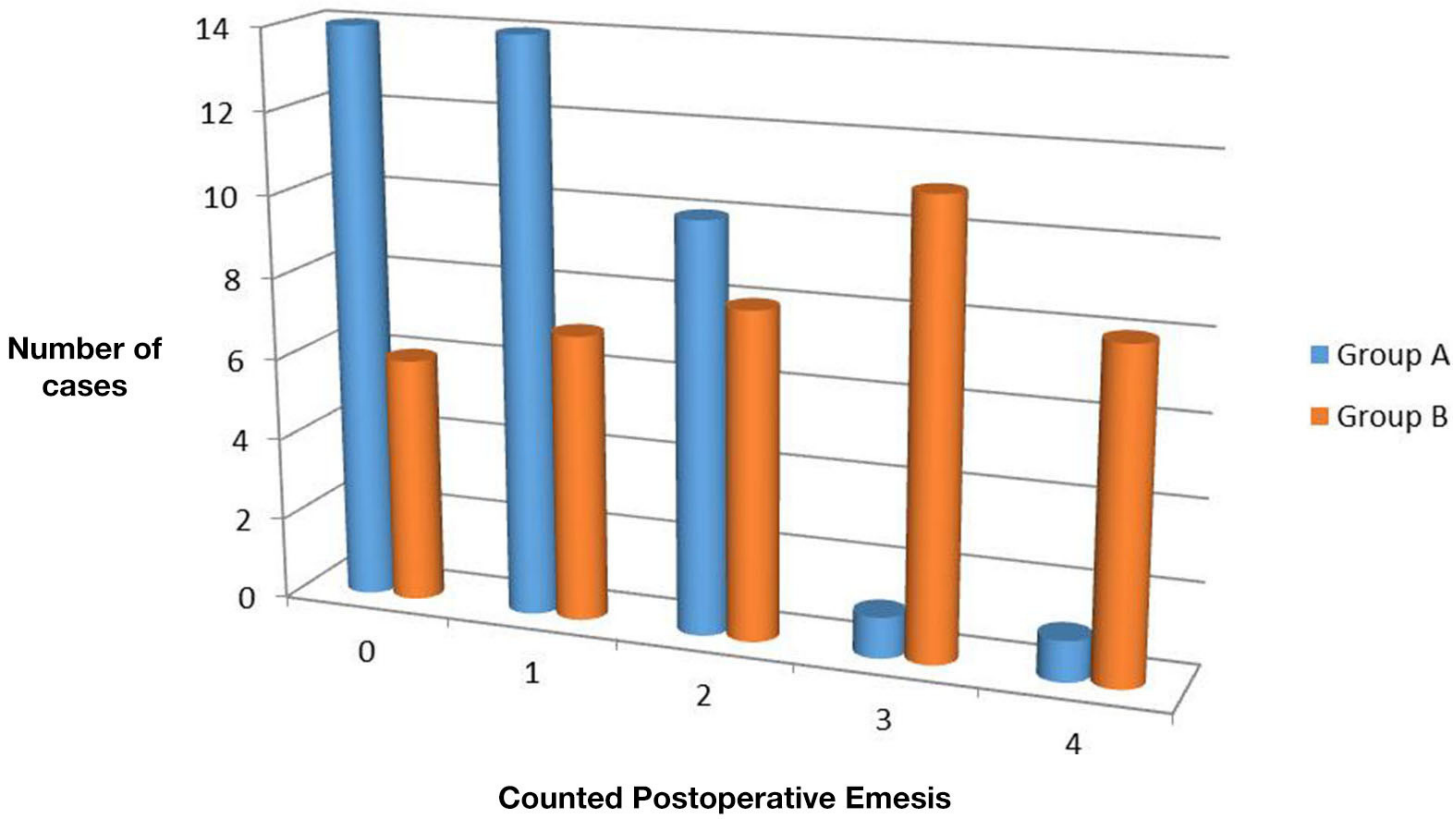
Number of times of post-operative emesis during admission in both groups.

The time estimated to reach full feeding ranged from 12 to 24 h in group A (mean 15.17 ± 3.054 h). In group B, it ranged from 12 to 25 (mean, 18.8 ± 4.369 h). On statistical analysis, the difference was highly significant (*P* = 0) with shorter time in the laparoscopic group. Moreover, the number of times the baby vomited, during initiation of feeding post-operatively until reaching full feeding, ranged from 0 to 4 times in both groups. However, 0 or 1 emesis were reported in higher number of cases in group A, whereas more cases in group B showed 3 or 4 emesis attacks (Figure 3). The difference between both groups was statistically significant (*P* = 0.001).

**Figure 4:**
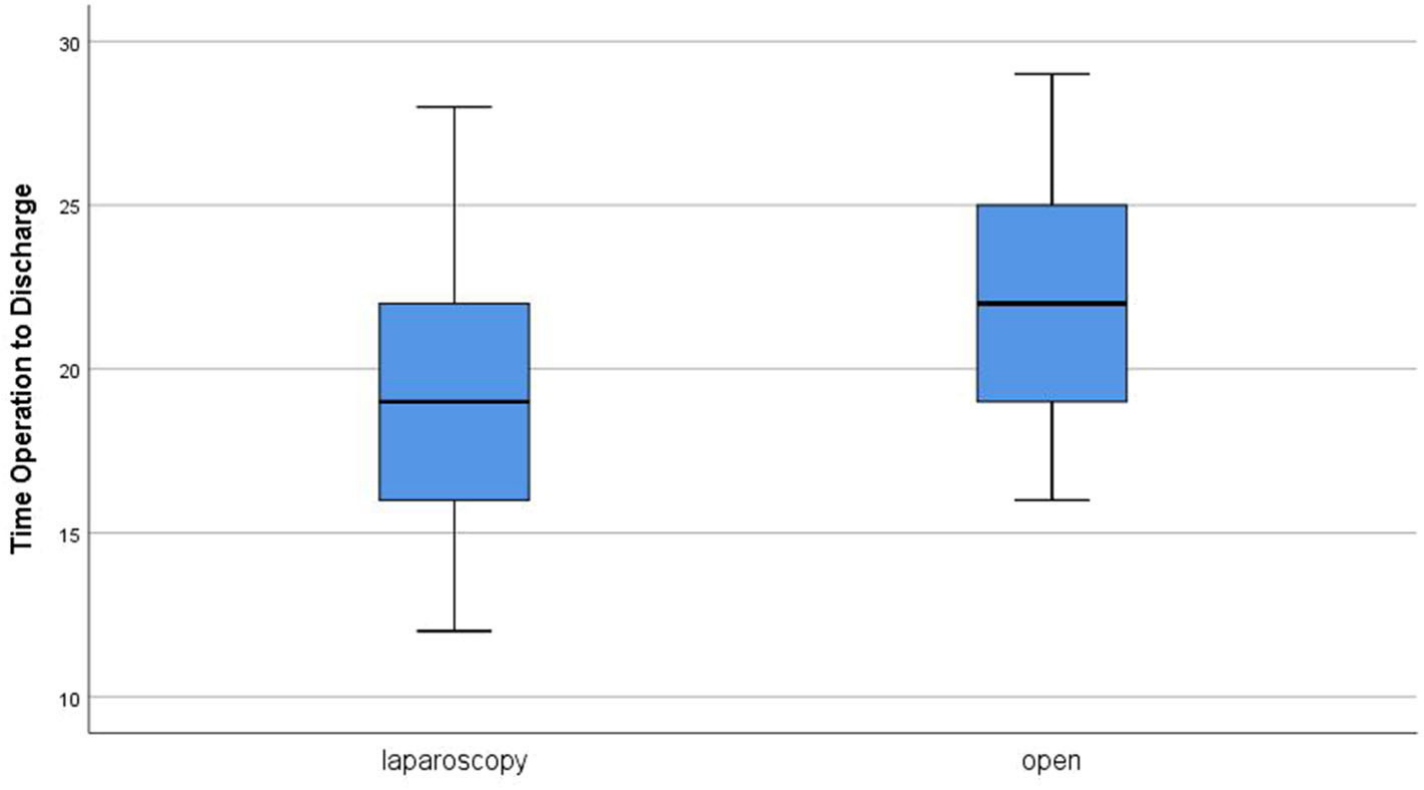
Time from operation to discharge in both groups.

**Figure 5:**
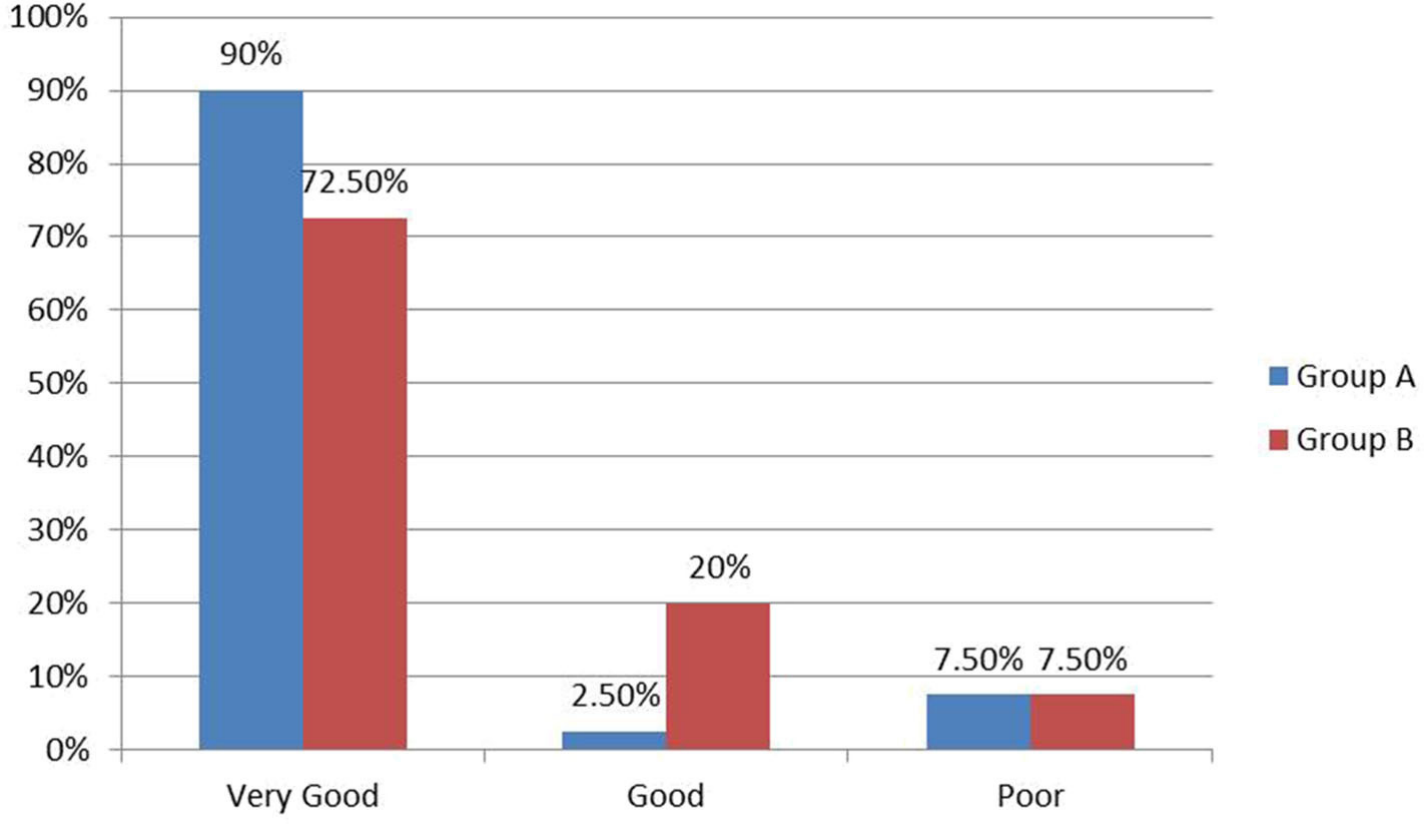
Parents' satisfaction about cosmetic outcome.

The time from operation to discharge ranged from 12 to 88 h with a median of 19 h in group A. In group B, it ranged from 16 to 29 h, and the median was 22 h (Figure 4). Comparing the two groups revealed a statistically significant difference in favor for group A (*P* = 0.004). Parents reported very good cosmetic outcome in 90% of cases after laparoscopy vs. 72.5% in the open group with a statistically significant difference in comparison (*P* = 0.045) (Figure 5). The three cases with poor cosmetic outcome in the laparoscopic group were all subjected to open approach for management of complications.

Incomplete pyloromyotomy occurred exclusively in group A as it was reported in two cases (5%), and both managed by redo OP. However, there was no statistically significant difference between both groups regarding such complication (*P* = 0.152). Other post-operative complications included wound infection in one case in group B (2.5%). Nevertheless, on using the Clavien–Dindo classification of surgical complications (14) for pooling the severe complications, group A showed five cases with grade IIIb complications that required interventions under general anesthesia, whereas group B were devoid of such grade of complications, with the difference now being statistically significant (*P* = 0.02) (Table 2).

**Table 2:** Surgical complications according to the Clavien–Dindo Classification.

**Grade**	**Group A**	**Group B**
**I (No intervention)**	0	Two cases (one controlled bleeding, one wound infection)
**II (Need pharmacological treatment)**	0	0
**III (Need surgical intervention)**	Five cases (two mucosal perforation, two incomplete pyloromyotomy, one laparotomy for a suspected perforation)	0
**IV (Life threatening)**	0	0
**V (Death)**	0	0

## Discussion

Although OP stood the test of time and remained the standard procedure for IHPS for many years, LP is widely spreading competing with the traditional technique. In the United States, performing LP significantly increased from 59% in 2013 to 65.5% in 2015 (*P* < 0.001) (15). The outcome of both approaches was compared by several studies and meta-analyses with contradictory results regarding the advantages and disadvantages of each technique (16–18). Currently, there are no clear evidence- based recommendations, and the selection between both approaches is still directed by the surgeons' preference.

In the present study, the laparoscopic approach was found faster than the open one with a highly significant statistical difference (*P* = 0). This agrees with several other reports (19, 20). However, Hall et al. (21) performed a meta-analysis that included eight studies and 595 cases and concluded no significant difference regarding the operative time between laparoscopic and open techniques. This may be because the operative time was defined, similar to our study, as the time from incision to dressing in only two of the studies included in the meta-analysis, and it was not clearly defined in the other six studies. On the other hand, Oomen et al. (22) reported a median operative time of 40 min in laparoscopic group vs. 33 min in the open group with a highly significant difference (*P* = 0) in the favor on the open technique. However, this study aimed at evaluation of the learning curve in pediatric laparoscopy by comparing early and late cases regarding complications. Thus, the long operative time in the laparoscopic group can be assumed to the earlier cases. Similarly, Leclair et al. (8) reported shorter operative time after OP and attributed this difference to the lack of experience in LP.

Binet et al. (5) noted that the operative time significantly affected the time needed to reach full feeding (*P* = 0.006). In this study, LP was found to be followed by shorter time to reach full feeding, less post-operative emesis, and shorter hospital stay. This agrees with the results of Sola and Neville (16); however, there were conflicting conclusions in other studies. In three meta-analyses conducted in 2004, 2012, and 2017, there is agreement with the shorter time to reach full feeds after laparoscopy. However, shorter hospital stay after laparoscopy was documented only in the first one, whereas there was no significant difference between laparoscopic and open techniques in the later studies (17, 18, 21). Other studies reported no significant difference even in the time to reach full feeds (7, 8, 20). These data suggest that the shorter time to reach full feeds after LP may be related to the shorter operative time, which was not always significantly different than open approach in different studies.

Cosmetic satisfaction and less post-operative pain are usual advantages of laparoscopic surgery. Moreover, there were some reports of a worse body image and poor cosmetic satisfaction after OP (10). Laparoscopic pyloromyotomy resulted in less post-operative pain and better cosmetic outcome and parents' satisfaction in the current study. Similar conclusions reported by Rumsey and Harcourt (23) and Binet et al. (5). St Peter et al. (24) evaluated the cosmetic outcome in more objective manner by asking the parents to complete a validated Patient Scar Assessment Questionnaire with photos. This study illustrates with statistical significance that scars after laparoscope are less abundant than scars from open procedure, and also the laparoscope has better patient and parent satisfaction and aesthetic results. They also noted that lower doses of analgesics required after LP.

In the present study, there was no significant difference between LP and OP regarding each type of complications. However, the only major complications reported in this study, mucosal perforation and incomplete pyloromyotomy, occurred exclusively after LP, which raised serious concerns. Sathya et al. (18), after systematic review of nine studies and meta-analysis of five studies, concluded no significant difference between both techniques in major perioperative complications (*P* = 0.74). Nevertheless, on specifically comparing incomplete pyloromyotomy, a 4% higher rate was reported after LP, which was significant (*P* = 0.03). In an earlier systematic review, there was no significant difference in both mucosal perforation and incomplete pyloromyotomy, but the difference approached the significance in incomplete pyloromyotomy (*P* = 0.06) (17). Hall et al. (25), in one of the largest series of pyloromyotomy (2,830 cases), concluded that LP was a marginally significant predictor of incomplete pyloromyotomy (*P* = 0.046) but not of mucosal perforation (*P* = 0.153). Furthermore, after pooling major complications in the present study, the difference between both groups became statistically significant, and such pooling was not performed in most of the previous studies and meta-analyses. This adds more concerns that will need further evaluation.

In the present study, all cases of LP were operated on by surgeons whose experience ranged from previous 25 to 75 LPs. Van Der Bilt et al. (26) studied the impact of learning curve on the outcome of LP by comparing the results in patients operated on from 1993 to 1996 and those operated on from 1996 to 2002. They concluded that the operative time decreased significantly for all surgeons after 15 pyloromyotomies. Moreover, the incidence of mucosal perforation dropped from 8.3 to 0.7% and incomplete pyloromyotomy declined from 8.3 to 2.7%. Yet, Oomen et al. (22) reported a cutoff of 35 procedures is needed to reach a plateau in the learning curve with a significant decrease in complications.

Limitations of the current study should be admitted. First, there was some difference in laparoscopic experience among surgeons who performed LP, and the correlation between the level of experience and the incidence of complications was not studied. Second, two different techniques was used to initiate the gastric incision in LP with no clear data about the impact of this step on the incidence of complications. However, another study is going in our institution with initial results suggesting no significant difference between the use of hook diathermy and knife regarding the outcome of LP. Third, the traditional upper transverse incision was preferred over the more cosmetic supraumbilical incision in open cases to reduce variations between surgeons as a confounding factor that may affect the comparison. Although this provided standard conditions for comparison of the primary outcomes, it mostly affected comparison of the cosmetic results in favor of the laparoscopic group. At last, further studies on a larger number of cases may be required for more accurate conclusions.

## Conclusion

Laparoscopic pyloromyotomy was found superior to open approach regarding faster operative time, less need of analgesics, easier development of oral feeding, shorter hospital stay, and better parents' satisfaction. Yet, there are still some concerns about the safety and efficiency of this procedure over open technique. However, these concerns are losing ground with the progress of the learning curve of the laparoscopic approach.

## Data Availability Statement

The datasets used and/or analysed during the current study are available from the corresponding author on reasonable request.

## Ethics Statement

The studies involving human participants were reviewed and approved by Institutional Research Board—IRB Faculty of Medicine—Mansoura University. Written informed consent to participate in this study was provided by the participants' legal guardian/next of kin.

## Author's Note

This paper was presented at the 9th Annual Congress of the European Society of Pediatric Endoscopic Surgeons, September 11th–13th, 2019, Vicenza, Italy.

## Author Contributions

II shared in designing the study protocol, was a major contributor in collection and analysis of data, and shared in writing the manuscript. RE shared in collection of data, statistical analysis, and revision of the manuscript. AE shared in designing the study protocol and revision of the results and the manuscript. KA shared in revision of the results and the manuscript. HS shared in designing the study protocol and was a major contributor in analysis of data and in writing the manuscript. All authors contributed to the article and approved the submitted version.

## Conflict of Interest

The authors declare that the research was conducted in the absence of any commercial or financial relationships that could be construed as a potential conflict of interest.

## Abbreviations

IHPS: Infantile hypertrophic pyloric stenosis
LP: Laparoscopic pyloromyotomy
OP: Open pyloromyotomy
RCTs: Randomized controlled trials
PAIN: Pain Assessment In Neonates
SPSS: Statistical Package for Social Science.
